# Metastasis of a prostate adenocarcinoma to mandible: A case report and review of literature

**DOI:** 10.1002/ccr3.3065

**Published:** 2020-08-07

**Authors:** Mahboube Hasheminasab, Abbas Karimi, Mehdi Kardoust Parizi, Farid Kosari, Amirali Asadi

**Affiliations:** ^1^ Oral and Maxillofacial Surgeon Craniomaxillofacial Research Center Tehran University of Medical Sciences Tehran Iran; ^2^ Department of Oral and Maxillofacial Surgery School of Dentistry Tehran University of Medical Sciences Tehran Iran; ^3^ Department of Urology Shariati Hospital Tehran University of Medical Sciences Tehran Iran; ^4^ Department of Pathology Shariati Hospital Tehran University of Medical Sciences Tehran Iran; ^5^ Resident of Oral and Maxillofacial Surgery School of Dentistry Tehran University of Medical Sciences Tehran Iran

**Keywords:** adenocarcinoma, mandible, metastasis, prostate

## Abstract

Dentists and physicians should include oral metastases originating from prostate adenocarcinoma as a rare differential diagnosis of jaw lesions that can produce periosteal reactions in the radiographic features such as osteosarcoma.

## Graphical abstract

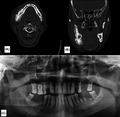

## 1 INTRODUCTION

Carcinoma of prostate accounts for 25% of all malignancies in men^1^ that tends to metastasize to bone. Ribs, ilium of pelvis, the vertebral column, and skull are often involved, whereas <1% of malignant tumors metastasize to maxillofacial region.^1^ Bone metastasis in oral cavity is extremely rare and represents 1% of all malignant oral neoplasia. The incidence of prostate cancer metastasis in the maxillofacial region is 80 to 90% in mandible, mainly in molar region, and is always a sign of spreading of the cancer.^2^

In this paper, we present the details of a 64‐year‐old male patient with mandibular metastasis from advanced prostate adenocarcinoma. In addition, we have reviewed 10 case reports of metastatic prostate adenocarcinoma to mandible in the literature.

## 2 CASE PRESENTATION

A 64‐year‐old man was referred to the department of oral and maxillofacial surgery complaining of a mass on the right side of mandible associated with paresthesia of the right side of lower lip.

**Figure 1:**
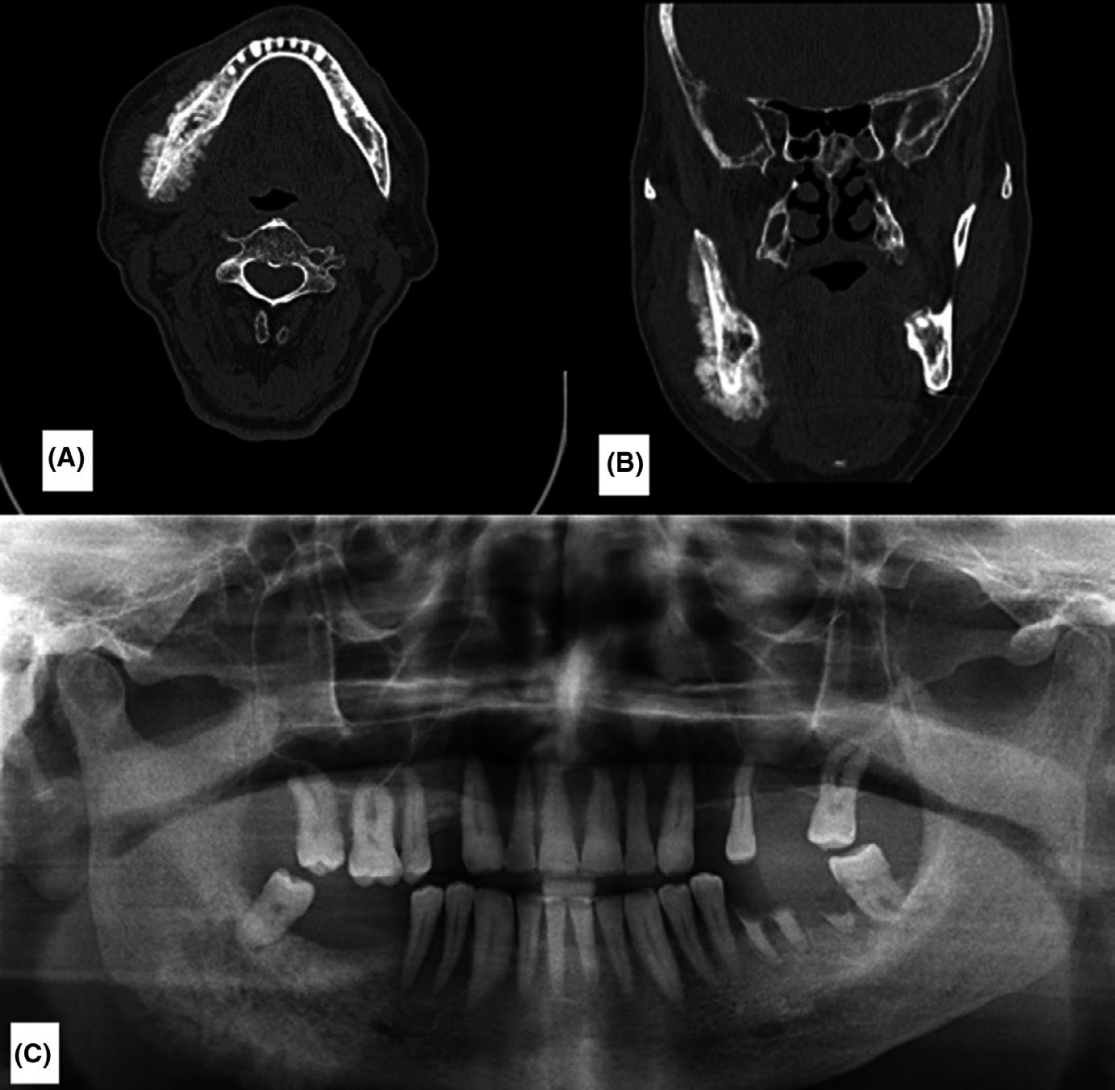
A and B, Axial and Coronal view show a sunray spicules/sunburst appearance of periosteal reaction in the right posterior body, angle, and ramus of mandible mimicking osteosarcoma variable with both bone destruction and bone formation. C, Panoramic view

**Figure 2:**
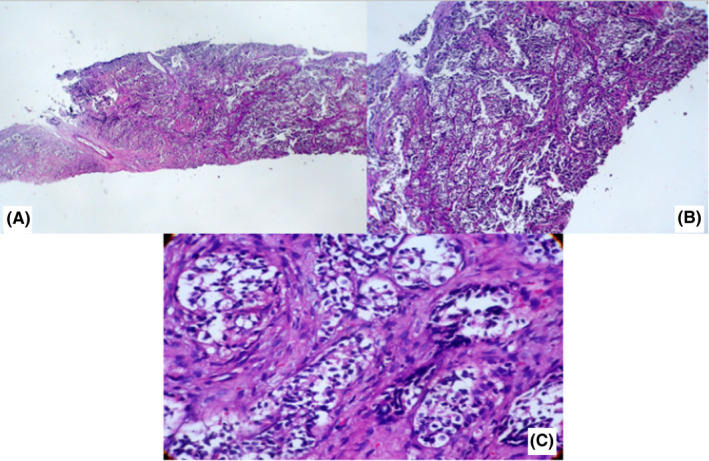
The biopsy sample was taken in Amiralam hospital, Tehran, Iran, A, low power view of the tumor showing fibro‐connective tissue with dense infiltration by nests of tumor cells (H&E ×40), B, The tumor cells form solid nests and glandular structures, separated by thin fibrous septa (H&E ×100), C, The Individual tumor cells in the nests have small round to medium‐sized oval hyper chromatic nuclei and moderate amount of clear cytoplasm Mitotic figures are infrequent. No necrosis is seen (H%E ×400)

Intraoral examination revealed little expansion of right mandible with bony hard consistency measuring about 5 cm, which caused facial asymmetry. CT scan images showed a lesion with periosteal reaction along with bone destruction and bone formation in the ramus of right mandible without perforation of cortical table and mandibular canal destruction (Figure 1). He has undergone incisional biopsy of the right jaw lesion and the result reported “clear cell carcinoma” which may primarily originate from genitourinary tract such as kidney, bladder, and prostate gland (Figure 2). According to elevated serum prostate‐specific antigen (PSA) levels, needle biopsy was obtained from prostate. Histopathologic analysis of prostate biopsy confirmed prostate adenocarcinoma of Gleason grade 5 + 5 = 10. IHC staining was positive for CK (AE1/AE3) and PSA. The patient had also elevated level of serum alkaline phosphatase (386 IU/L), which suggested escalated bone metabolism. After revision of jaw specimen, which was taken in Amiralam hospital, Tehran, Iran, the diagnosis of metastatic adenocarcinoma was confirmed. The patient underwent bilateral orchiectomy as a palliative management with dramatic primary response.

## 3 DISCUSSION

Prostate cancer typically metastasizes to bones, such as lumbar vertebrae, thoracic vertebrae, and the pelvis. Metastasis of prostate cancer to the maxillofacial region is relatively rare.^3^

Because metastatic lesions to the jaw mimic other oral lesions, diagnosis is a dilemma for dentists. These lesions may cause paresthesia, pain, ulcers, swelling, and pathologic fracture, and these symptoms may be mistaken with other oral lesions. Imaging, histopathologic examination, and patient's history of cancer would help the diagnosis. Also for the definitive diagnosis, scintigraphy and IHC panel may help the clinician.^4^, ^5^ However, most patients with oral metastasis generally have the primary cancer well diagnosed. In a case reported by Menezes et al, radical prostatectomy was done for the patient 3 years before mandible metastasis and because patient's chief complaint which was pain and paresthesia, he was on neurologic drugs with a diagnosis of trigeminal neuralgia.^2^ Metastatic tumors of the head and neck are most commonly located in the mandibular molar region.^3^ The posterior mandible is the most susceptible metastasis site because of its rich blood supply.^6^

Metastases cases of mandible reported in the literature were mostly in angle and body region and rarely in condylar area. Most frequently, patients were in their 7th and 8th decades of life.^2^, ^7^, ^8^, ^9^ The most common chief complaints of the patients were pain and swelling, and other manifestations such as paresthesia, limited mouth opening, and preauricular pain were also reported.^3^, ^8^, ^10^

Radiographic feature showing periosteal reactions can be classified as single layer, multilayered, solid, speculated, perpendicular, sloping, complex, Codman triangle, and sunburst.^11^ The appearance of a “sunburst” periosteal reaction is suggestive of rapid onset pathology,^6^ and in this case, it highly suggests a malignant bone forming tumor, such as an osteosarcoma.

Table 1 reviews previous cases of metastasis to mandible in the literature.

**Table 1:** reported cases of metastatic prostate adenocarcinoma

No.	Author(s)	Age	Symptoms	Location	Side
1	Taghavi et al 2016	82	Pain and Paresthesia	Angle of mandible	Right
2	Lee et al 2016	80	Numbness and swelling	Angle of mandible	Left
3	Takahashi et al 2014	60	Swelling	Angle of mandible	Left
4	Orhan et al 2014	78	Numbness and pain	Molar region to TMJ area	Left
5	Menezes et al 2013	54	Pain and Paresthesia	Molar and premolar region	Left
6	Freudlsperger et al 2012	75	Pain and limited mouth opening	Condyle of mandible	Left
7	Habal et al 2011	68	Pain and swelling	Angle and body of mandible	Left
8	Saijo et al 2008	75	preauricular pain	Condyle of mandible	Left
9	Halachmi et al 2000	71	Reduced sensation of chin	Not available	Left
10	Iga et al 1997	76	Swelling	Molar region	Left

As seen in the table, common clinical features among most cases were pain, paresthesia, and swelling. Posterior of mandible from body to angle and condyle were the common sites for metastasis. In most of the cases reviewed, left side was involved.

Differential diagnosis for this case can be osteosarcoma which can show “sunburst” periosteal reaction.^12^ Other cancers such as Ewing's sarcoma, chondroblastoma, eosinophilic granuloma, osteoid osteoma, leukemia, and lymphoma can also mimic same clinical and radiographic features.^13^

In conclusion, dentists and general physicians should include oral metastases originating from prostate adenocarcinoma as a rare differential diagnosis of jaw lesions that can produce periosteal reactions. Metastases are more common in elderly people and more common in posterior of mandible. The patient's chief complaint is usually pain and swelling with paresthesia.

## CONFLICT OF INTERESTS

None.

## AUTHOR CONTRIBUTIONS

MH: conceived of the presented data. AK: carried out the experiment; MKP and FK: contributed to sample preparation; AA: wrote the manuscript in consultation with other authors.
